# Mid‐term outcomes in patients younger than 65 years undergoing reverse total shoulder arthroplasty demonstrate favourable functional results with high rates of return to activity and work

**DOI:** 10.1002/jeo2.70820

**Published:** 2026-07-02

**Authors:** Lorenz Fritsch, Colby Wollenman, Michael Nocek, Marilee Horan, Brendon Mitchell, Grant Dornan, Alan Villegas Meza, Maximilian Hinz, Marco‐Christopher Rupp, Peter Millett

**Affiliations:** ^1^ The Steadman Philippon Research Institute Vail Colorado USA; ^2^ Department of Sports Orthopaedics Technical University of Munich Munich Germany; ^3^ The Steadman Clinic Vail Colorado USA

**Keywords:** age < 65, mid‐term follow‐up, return‐to‐activity, return‐to‐work, reverse total shoulder arthroplasty

## Abstract

**Purpose:**

The aim of the study was to evaluate outcomes, return‐to‐activity and work after reverse total shoulder arthroplasty in patients aged 65 years and younger. We hypothesised that patients would achieve functional improvement, with high rates of return to activity and return to work.

**Methods:**

A retrospective study was conducted for patients aged 65 years and younger who underwent primary reverse total shoulder arthroplasty (2011–2022) with a minimum 24‐month follow‐up. Outcomes included the American Shoulder and Elbow Surgeons score, pain visual analogue scale, Single Assessment Numeric Evaluation, Quick Disabilities of the Arm, Shoulder and Hand outcome measure, and 12‐Item Short Form Health Survey. Return to activity and work were also assessed with questionnaires. Complications, failures and correlations between age and outcomes were analysed.

**Results:**

Seventy‐two shoulders in 68 patients (80% of the original cohort) (mean age, 58.5 ± 8.5 years) were available at mean follow‐up of 6.2 ± 2.4 years. American Shoulder and Elbow Surgeons score (54.9 vs. 89.9), pain visual analogue scale (4 vs. 0), Single Assessment Numeric Evaluation (49 vs. 84), Quick Disabilities of the Arm, Shoulder and Hand outcome measure (40.9 vs. 13.6) and 12‐Item Short Form Health Survey Physical Component (39.6 vs. 51.1) improved postoperatively (*p* < 0.001). For the American Shoulder and Elbow Surgeons score, 80% met the minimal clinically important difference and 64% met the patient acceptable symptom state. Younger age correlated with higher post‐operative Single Assessment Numeric Evaluation scores (rho = –0.35, *p* = 0.003). No or minimal limitations in recreational and sportive activities were observed in 89% of patients. 72% returned to work within 4 weeks, and 95% reported no or slight work limitation, with greater difficulty among manual labourers. One failure (1%) and two complications (3%) occurred.

**Conclusion:**

Reverse total shoulder arthroplasty in patients aged 65 years and younger leads to improved functional outcomes and high patient satisfaction at mid‐term follow‐up. Furthermore, it enables high rates of return to both activity and work with low rates of complication and failure.

**Level of Evidence:**

Level IV.

**Abbreviations**
ASES: American Shoulder and Elbow Surgeons
BMI: body mass index
MCID: minimal clinically important difference
PASS: patient acceptable symptom state
PROMs: patient‐reported outcome measures
QuickDASH: The Quick Disabilities of the Arm, Shoulder, and Hand
RTA: return to activity
RTSA: reverse total shoulder arthroplasty
RTW: return to work
SANE: Single Assessment Numerical Evaluation
SF‐12 MCS: 12‐Item Short Form Health Survey – Mental Component Summary
SF‐12 PCS: 12‐Item Short Form Health Survey – Physical Component Summary
VAS: Visual Analogue Scale

## INTRODUCTION

Reverse total shoulder arthroplasty (RTSA) has emerged as a primary treatment option for several indications. These include rotator cuff arthropathy, massive irreparable rotator cuff tears, complex proximal humeral fractures, revision of anatomic shoulder arthroplasty, and glenohumeral osteoarthritis with severe bony deformity, and end‐stage intractable instability [7, 13, 17, 28, 32]. When appropriately indicated, RTSA has been shown to achieve significant improvements in pain and function [3] with durable results even at mid‐ to long‐term follow‐up [4, 19, 27].

Several factors, including sex, body mass index (BMI), age and history of prior shoulder surgery, have been identified as predictors of post‐operative outcomes and complication rates [1, 12, 18, 33]. Younger age, in particular, is frequently referenced as a risk factor for higher revision and complication rates, in comparison to older patient populations [8, 29, 35]. This observation may be grounded in a negative selection bias, given that younger individuals under consideration for RTSA often have more severe pathology and have exhausted other surgical options. Additionally, they may have higher functional demands compared to older patients, all of which increase the risk of revision [1, 24].

Although young age is a cited risk factor for poorer outcomes after RTSA, many young patients still achieve good and satisfactory results when appropriately indicated for RTSA [11, 16, 22, 23, 24].

When considering return to activity (RTA) and return to work (RTW)—parameters of substantial importance for the active individual—the available evidence in younger patient cohorts treated with RTSA remains limited [30, 31]. Furthermore, existing studies examining these outcomes following implantation of RTSA in younger cohorts are heterogeneous and often lack mid to long‐term follow‐up data [5, 11, 34].

The purpose of this study was to evaluate midterm clinical outcomes primarily assessed using the American Shoulder and Elbow Surgeons (ASES) score, failure rate and the factors influencing these outcomes in patients under the age of 65 undergoing RTSA. In addition, RTA RTW were evaluated in this population. We hypothesised that younger patients undergoing RTSA would demonstrate substantial improvements in functional outcomes, and exhibit high rates of RTA and RTW at midterm follow‐up, with low rates of failure.

## METHODS

This study was conducted with approval from the WCG IRB Clinical (WCG #20252021). All cases were performed by a single surgeon (PJM). Patients were prospectively and consecutively enroled into an institutional patient outcomes database. Based on these data, a retrospective cohort analysis was performed. Follow‐up was assessed using an electronic database. Inclusion criteria included any patient age 65 years or younger who underwent primary RTSA for osteoarthritis, end stage intractable instability, instability arthropathy or rotator cuff arthropathy between January 2011 and October 2022. Exclusion criteria were patient refusal to participate in research or prior arthroplasty. A clinical follow‐up of minimum 24 months was required.

### Surgical technique

The Delta Extend arthroplasty system (Depuy Synthes, Inc., Raynham, MA, USA; neck shaft angle 145**°**) was used for earlier cases; more recent cases were treated with the Arthrex Univers Revers Total Shoulder system—Apex Stem (Arthrex, Inc., Naples, FL, USA; neck shaft angle 135**°**). The Delta Extend arthroplasty system (Depuy Synthes, Inc., Raynham, MA, USA; neck shaft angle 145°) follows the principles of the classic Grammont design. Its center of rotation is medialized and inferiorized, and the implant features a relatively medialized humeral configuration. In contrast, the Arthrex Univers Revers Total Shoulder system—Apex Stem (Arthrex, Inc., Naples, FL, USA; neck shaft angle 135°) represents a lateralized reverse shoulder arthroplasty concept, offering greater options for both glenoid and humeral lateralisation. This is facilitated by its 135° neck‐shaft angle and its inlay humeral design, which allow restoration of a more lateral center of rotation. The patient was positioned in a slightly inclined beach‐chair position, and the operative arm was stabilised using an arm holder. A standard deltopectoral approach was utilised. Careful dissection was carried down to the joint capsule, and the subscapularis tendon was released using a peel‐off technique. Whenever possible the subscapularis tendon was preserved for later repair otherwise tenotomy was performed. The axillary nerve was routinely identified and protected.

Following dislocation of the humeral head, the humeral head osteotomy was performed, and the humeral shaft was prepared by reaming and broaching. The glenoid was then exposed, the labrum and biceps were removed. Glenosphere size was determined intraoperatively after preparation of the glenoid. An appropriately sized glenosphere trial was then used prior to reduction to ensure optimal fit and stability. In all patients treated with the Arthrex Univers Revers Total Shoulder system—Apex Stem (Arthrex, Inc., Naples, FL, USA), standardised +4 mm lateralisation was applied. This configuration was selected based on evidence demonstrating improved outcomes when combined with a 135° neck‐shaft angle [2, 20, 25].

Final humeral preparation was carried out, and appropriately sized stem, cup, and inlay components were implanted according to the manufacturer's instructions. If the subscapularis was deemed repairable, trans‐osseous sutures were placed around the humeral stem for subsequent subscapularis repair which was then performed using a modified Mason‐Allen technique. Vancomycin powder was applied to the shaft area.

The shoulder was reduced, and intraoperative stability was assessed. Final implants were inserted and the RTSA was reduced. Stability was carefully assessed. The wound was then closed in standard fashion.

### Rehabilitation

Immobilisation in a shoulder abduction sling was required for 3 weeks, allowing only passive range‐of‐motion exercises during this period. Active and active‐assisted range of motion was initiated after 3 weeks postoperatively. In cases where the subscapularis was repaired, external rotation was limited to 30 degrees for approximately 3–4 weeks postoperatively.

### Outcome measurement

Patient‐reported outcome measures (PROMs) were collected preoperatively and at a minimum follow‐up of two years postoperatively. The American Shoulder and Elbow Surgeons (ASES) score, Single Assessment Numeric Evaluation (SANE), Disabilities of the Arm, Shoulder, and Hand (QuickDASH), and the Physical Component Summary (PCS) of the 12‐Item Short‐Form Health Survey (SF‐12) were obtained. Additionally, a visual analogue scale (VAS) for pain and a 10‐point Likert scale assessing subjective satisfaction—where 10 represented maximum satisfaction—were recorded.

The percentage of patients reaching the minimal clinically important difference (MCID) and the patient acceptable symptom state (PASS) thresholds were evaluated using established cutoffs: 11.4 for the minimally clinically important difference and 80.8 for the PASS for the ASES score, respectively [6].

The primary outcomes were the ASES score, as well as achievement of the MCID and PASS for the ASES score. Secondary outcomes included the SANE score, the QuickDASH score, and the SF‐12 PCS score, VAS for pain and subjective satisfaction.

Also, correlation between age, prior rotator cuff surgery, and outcome scores was assessed. Range of motion was adapted from visitation reports and assessed separately during clinic visits. Range of motion was assessed by a physician preoperatively and at the most recent follow‐up examination, which occurred at least four months after surgery.

### RTA

RTA was assessed using a custom questionnaire at a minimum of 24 months postoperatively. Patients were asked about their ability to participate in employment‐related, recreational, and sports activities and if they participated in a main sport discipline. Four response categories were provided: no impairment, somewhat difficult to participate in regular recreational or sports activities, very difficult, or unable to participate. If eligible, patients could voluntarily report the level at which they were able to compete or participate in their primary sport compared with their pre‐injury level. Furthermore, patients were asked whether they experienced pain during activities of daily living or recreational activities. Both pain questions were rated on a four‐point scale consisting of no pain, mild pain, moderate pain, and severe pain.

Patients were also asked whether they had modified their main sport and whether they had modified their fitness programme. Modifications to the fitness programme were categorised as yes, no, somewhat modified or not applicable. Also, they were asked for reasons why they modified their sporting activity.

### RTW

Work was considered manual labour if it involved regular physical labour with repetitive upper extremity use. Patients completed a custom return‐to‐work questionnaire in which they were asked about their time of RTW: immediate return, return within 4 weeks, or return after 4 weeks. The questionnaire was administered at least 24 months postoperatively. They were further asked about the level of pain experienced at work, selecting from four categories: no pain, mild pain, moderate pain or severe pain. In addition, patients rated how their shoulder affected their ability to work, indicating whether their shoulder caused no limitation, slight limitation, major limitation or prevented them from working entirely. Finally, patients were asked to specify the reason for any temporary work restrictions.

### Failures and complications

Failure was defined as any revision surgery involving complete revision, change of the arthroplasty components or revision due to infection. Complications were defined as any arthroplasty‐related event managed either conservatively or surgically but not requiring component revision.

### Statistical analysis

All statistical analyses were performed using SPSS software (version 30.0; IBM Corp., Armonk, NY, USA). Categorical variables are reported as frequencies and percentages. Normality of continuous variables was assessed with the Kolmogorov–Smirnov test and confirmed by visual inspection. Normally distributed data are presented as mean ± standard deviation, while non‐normally distributed data are summarised as median with interquartile range (25th–75th percentile). Wilcoxon signed‐rank tests were used to compare baseline and post‐operative PROMs within subjects due to the non‐normal distribution of the outcome variables. Associations between continuous variables were evaluated using Spearman's rho correlation coefficients. Statistical significance was defined as *p* < 0.05.

## RESULTS

Follow‐up was obtained for 72 shoulders from 68 unique patients (80%; follow‐up was not available for 18 shoulders (20%). Of these, 41 patients (60%) were male. The mean age at the time of surgery was 58.5 ± 8.5 years. Mean follow up was 6.2 ± 2.4 years after surgery. Further surgical and demographic details are presented in Table 1.

**Table 1:** Surgical and demographical results.

Variable	Value
Sex *n* (%)	
Male 41 (60%)	
Female 27 (40%)	
Age at surgery (years, mean ± SD)	58.9 ± 8.3 (range 22–64.8 years)
Follow‐up (years, mean ± SD)	6.2 ± 2.4 (range 2–12.9 years)
Previous rotator cuff surgery *n* (%)	26 (36%)
Combined osteoarthritis and rotator cuff arthropathy *n* (%)	45 (63%)
Rotator cuff arthropathy *n* (%)	21 (29%)
End stage instability *n* (%)	4 (6%)
Instability arthropathy *n* (%)	1 (1%)
Severe glenoid wear and retroversion *n* (%)	1 (1%)
Dominant shoulder affected *n* (%)	42 (58%)
Use of a 135° shaft *n* (%)	41 (57%)
Glenosphere size (median, interquartile range, range)	39 (36–39, range 33–39)
Lateralisation *n* (%)	
+4	36 (89%)
+2	1 (2%)
No lateralisation	4 (9%)
Stem size (median, interquartile range, range)	8 (6–9; range 5–11)
Use of a 145° shaft *n* (%)	31 (43%)
Glenosphere size (median, interquartile range, range)	38 (38–38, range 38–42)
Lateralisation *n* (%)	
No lateralisation	31 (100%)
Stem size (median, interquartile range, range)	12 (10–14, range 8–16)

*Note*: Values are presented as mean ± standard deviation or number (percentage). Values are presented as median (interquartile range) for non‐normally distributed data. Below the number of procedures performed with each prosthesis system, general information on the components of the respective implant is presented.

### PROMs

All PROMs (ASES, Pain [VAS], SANE score, QuickDASH and SF‐12 PCS) showed significant improvement from preoperatively to postoperatively (Table 2). Regarding ASES, 64% of patients achieved the PASS, and 80% of patients achieved the MCID. Median satisfaction was 8 (3–10).

**Table 2:** Patient‐reported outcome measures (PROMs).

Parameter	Pre‐operative	Post‐operative	*p*‐value
ASES	54.9 (38.3–68.3)	89.9 (73.3–94.9)	**<0.001**
Pain (VAS)	4 (2–6)	0 (0–1)	**<0.001**
SANE score	49 (27–59.5)	84 (49–89)	**<0.001**
QuickDASH	40.9 (30.7–55.7)	13.6 (6.8–38.6)	**<0.001**
SF‐12 PCS	39.6 (34.1–44.8)	51.1 (37.8–55.8)	**<0.001**
Forward flexion	125° (80°–160°)	140° (120°–160°)	**0.002**
External rotation	38.2° ± 18.5°	43.2° ± 15.5°	0.22
Abduction	93.9 °± 46.5°	108.5° ± 32.3°	0.25

*Note*: Values are presented as mean ± standard deviation for normally distributed data. Values are presented as median (interquartile range) for non‐normally distributed data. A *p*‐value < 0.05 was considered statistically significant. Bold values indicate statistically significant. Abduction was only assessed in 53 participants (73%).

Abbreviations: ASES, American Shoulder and Elbow Surgeons Standardised Shoulder Assessment Form; MCS, Mental Component Summary; PCS, Physical Component Summary; QuickDASH, Disabilities of the Arm, Shoulder and Hand; SANE, Single Assessment Numeric Evaluation; SF‐12, 12‐Item Short Form Survey; VAS, Visual Analogue Scale.

No significant differences in PROMs were found between the different arthroplasty models (*p* > 0.05). In the pre‐operative comparison, correlations between age and pre‐operative scores showed a non‐significant trend toward lower QuickDASH (rho = –0.08, *p* = 0.5), higher SANE (rho = 0.16, *p* = 0.2), and ASES scores (rho = 0.15, *p* = 0.2) in older patients.

Correlation analysis demonstrated no significant association between age at the time of surgery and post‐operative outcomes for most PROMs. In addition, prior rotator cuff surgery did not affect the outcomes. However, age at surgery showed a significant negative correlation with the post‐operative SANE score (rho = –0.35, *p* = 0.003) indicating higher post‐operative SANE scores in younger patients. Additionally, a weak negative correlation was identified between age and the SF‐12 PCS score (rho = –0.28, *p* = 0.02).

#### RTA

**Figure 1:**
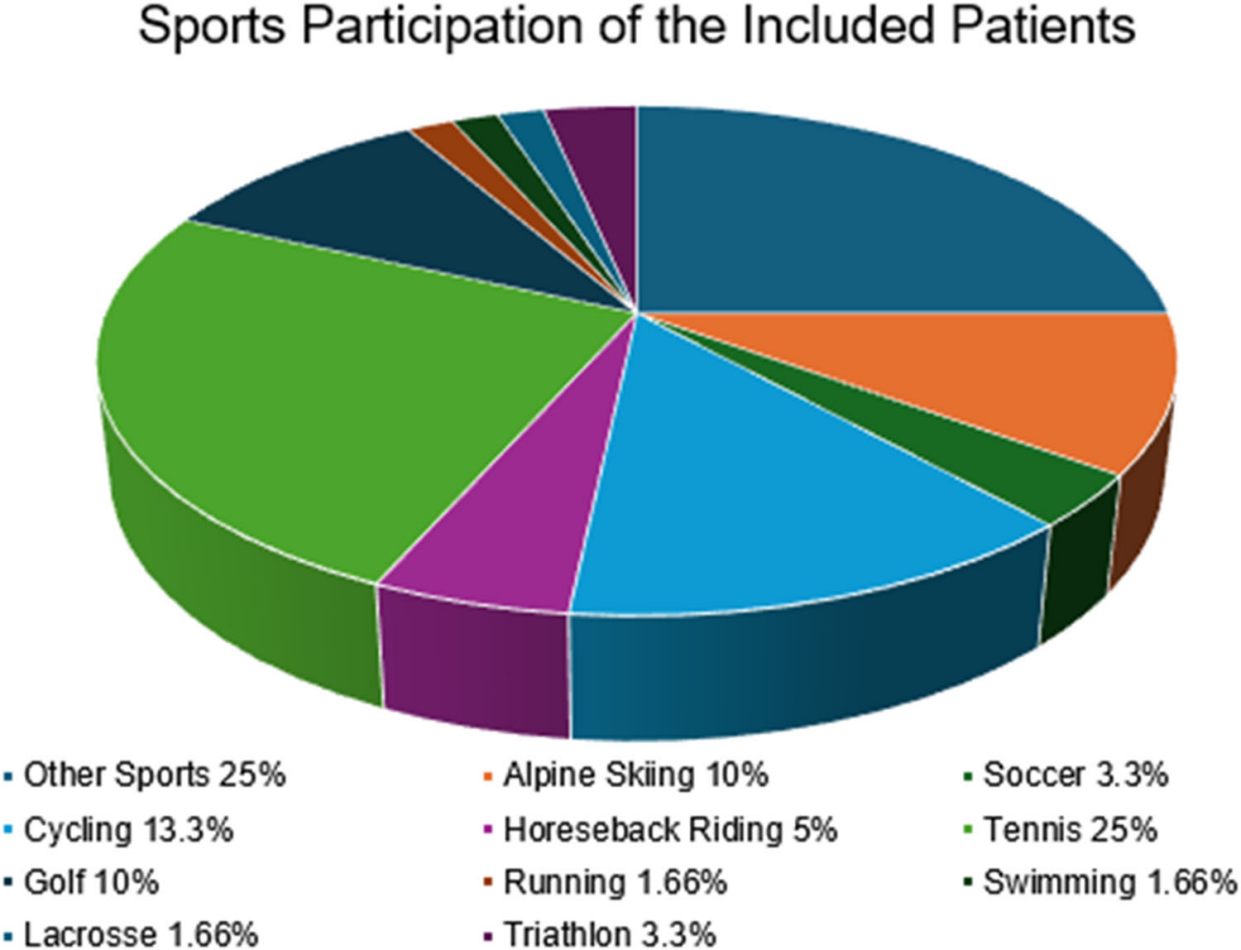
Distribution of the primary sport among the included patients, expressed as percentages of the study cohort.

Tennis was the most common primary sport, representing 25% of patient participation. The detailed distribution of sports is shown in Figure 1.

The primary sports occupations of the included patients are shown in Figure 1. Regarding symptoms and function at the end of follow up, 78% of patients reported no or only mild pain during daily activities, and 75% reported no or only mild pain during recreational activities. Additionally, 89% were able to perform sport normally or with only slight impairment, and 89% could participate in recreational activities without or with only minimal restriction.

Overall, 49% of patients responded to the question regarding competitive ability. Of those, 53% were able to compete at ≥75% of their previous level. Among these, the competitive sports comprised skiing, soccer, cycling, horseback riding, tennis, golf and lacrosse. Among those who were no longer able to compete, the previously practiced sports included cycling, skiing, and tennis. Additional data is presented in Table 3.

**Table 3:** Return‐to‐activity.

Impairment in recreational activity	*n* (%)
No impairment	44 (62%)
Somewhat difficult	19 (27%)
Very difficult	4 (6%)
Unable	4 (5%)
Impairment in sporting activity	
No impairment	41 (59%)
Somewhat difficult	21 (30%)
Very difficult	3 (5%)
Unable	4 (6%)
Return to recreational competition	
Same or better level	8 (24%)
75%–99% of prior level	10 (29%)
50%–74% of prior level	6 (18%)
25%–49% of prior level	2 (6%)
<25% of prior level	2 (6%)
No longer competing	6 (18%)

*Note*: Summary of impairment in recreational and sporting activities and return‐to‐competition outcomes. Data are presented as absolute numbers (percentages).

#### Modification of activity

After surgery, a total of 20% of patients resumed their fitness activities without requiring any modifications to their training routine. Furthermore, 42% were able to return to their primary fitness programme, although minor adjustments were necessary.

**Figure 2:**
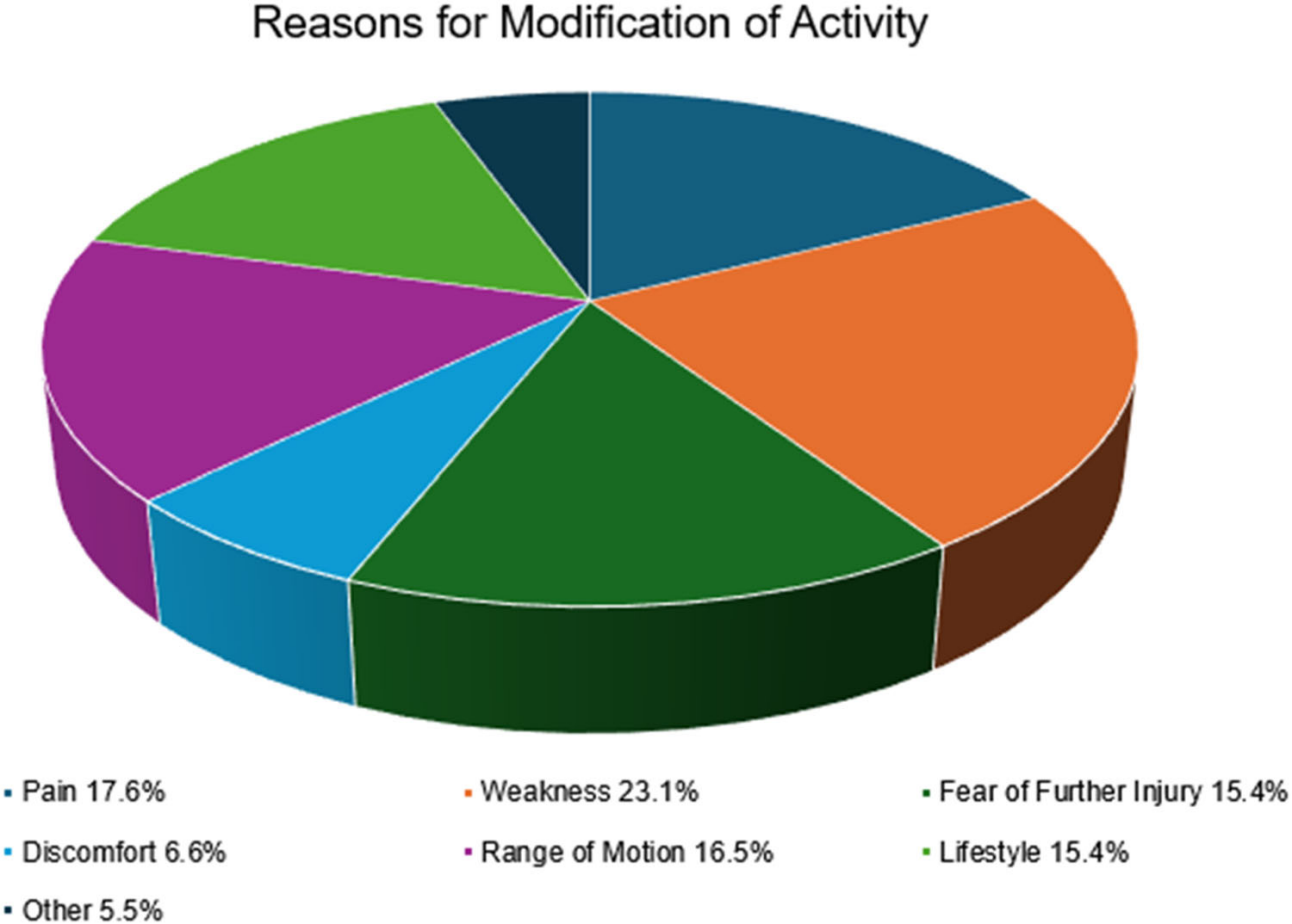
Percentage distribution of the reported reasons for activity modification, calculated relative to the frequency of responses. Weakness and pain during activity were the most commonly reported reasons for modifying activity.

Only 38% of patients required complete modification of their routine training programme. Reasons for the modification of their primary sport are presented in Figure 2.

#### RTW

RTW rate was 99%, one patient was already retired before surgery. In contrast, 5% of patients (mean age 60.1 ± 3.8 years) were unable to perform their job or reported major difficulty. Among these four patients, three patients reported severe pain at work, and one patient indicated that their shoulder function substantially limited their ability to work. Manual labour jobs accounted for 10% of occupations in our cohort. Of the four patients who reported difficulty working, two patients were employed in manual labour‐intensive occupations (construction and machinist). Further details are presented in Table 4.

**Table 4:** Return to work after RTSA in patients < 65 years.

Return‐to‐work timeframe *n* (%)	
No days missed	34 (28)
<4 weeks	19 (50)
>4 weeks	15 (22)
Pain during work	
No pain	45 (63)
Mild pain	12 (17)
Moderate pain	12 (17)
Severe pain	2 (3)
Impairment at work	
Normal work ability	52 (74)
Slight limitation	15 (21)
Major limitation	3 (4)
Unable to perform work	1 (1)

*Note*: Return‐to‐work outcomes after reverse total shoulder arthroplasty (RTSA) in patients younger than 65 years. Preoperatively, all patients were employed, except for one who was retired. One patient did not report the timeline of return to work. Values are presented as absolute numbers (percentages).

#### Failures and complications

Failures were observed in one patient (1%). In this case, a prosthetic dislocation occurred. The implant remained complication‐free for approximately 20 months; thereafter, the patient developed recurrent subluxations in certain positions, culminating in a complete dislocation triggered by a combined abduction and external rotation movement. The patient underwent revision surgery with a component exchange. The time between the index surgery and the revision procedure was 27 months.

One case of an acromial fracture (1%) was observed. The affected patient had known osteoporosis and sustained a fall, with the acromial fracture occurring as a consequence of the traumatic event, which was treated conservatively. This patient had no significant limitations in work, sports, or leisure activities. Additionally, one case of persistent post‐operative pain and shoulder stiffness affecting both activities of daily living and sports participation was noted (1%). The patient reported substantial impairment in work, sports, and recreational activities.

However, this complication was successfully treated with arthroscopic arthrolysis.

## DISCUSSION

The most important finding of this study is that patients younger than 65 years undergoing RTSA experienced significant functional improvement with high satisfaction, pain reduction and low complications. Further, a high percentage of patients were able to return to both activity and work with no to minimal impairment. While RTSA allowed for a rapid RTW, RTA often required modification, with the majority of patients being forced to change their primary sport. Another important finding was that younger age was not associated with inferior clinical outcomes but was instead associated with significantly higher post‐operative SANE scores.

An important point to emphasise is the role of specific neck‐shaft angle designs. In our cohort, we were not able to detect a significant difference in clinical outcomes between the different neck‐shaft angle designs used.

Prior studies have provided important evidence on this matter. A 135° neck‐shaft angle combined with a glenosphere with 4 mm of laterlaization has consistently been shown to improve outcomes, ROM and, importantly, to reduce scapular notching compared with higher‐angle designs [2, 20, 25].

Younger age has consistently been associated with higher revision rates following RTSA, likely reflecting increased activity demands, longer life expectancy, and greater surgical complexity [10, 16, 24, 35]. Goldenberg et al. reported revision rates of 5.8%–11.2% at mid‐term follow‐up, with complication rates of approximately 15%–18%, particularly among patients with prior shoulder surgery [16] Similarly, another study demonstrated that patients younger than 60 years had a threefold higher revision rate than older cohorts (5.2% vs. 1.8%) and lower satisfaction scores [24] Other literature reported lifetime revision rates of up to 30% when RTSA is performed between the ages of 46 and 50 years [35] The most common reasons for failure are dislocation/instability and component loosening [11, 16, 21]. Potential contributing factors include post‐traumatic arthritis, a higher number of prior surgeries, and the increased physical demands of younger patients [35] These data have historically led surgeons to defer RTSA to younger patients in favour of non‐operative or joint‐preserving strategies.

However, contemporary evidence demonstrates that younger patients can still achieve favourable and durable outcomes following RTSA. Several studies reported significant improvements in functional outcomes—ASES score (exceeding 80 points), Constant score, and VAS for pain. Simultaneously, no functional decline was noted [11, 23, 26]. Longer‐term data further support these findings. One study demonstrated that Constant scores improved from 24 to 59 and Subjective Shoulder Value from 20% to 71% at a mean 11.7‐year follow‐up in patients younger than 60, without late deterioration [11] However, it should be noted that overall survivorship at 10 years is approximately 76% [16] indicating a considerable risk of revision—particularly in patients younger than 65 years—who may require multiple revision procedures over their lifetime.

A unique finding of this paper comes from the granular assessment of impairment after a RTSA—particularly in sport and occupational performance—as this is frequently cited as a major concern in younger patient populations. The high rate of RTA demonstrated in this study, frequently with minimal to no impairment, aligns with prior, non‐age‐specific, return‐to‐sport literature. RTA rates have been previously reported at 77%–85% [14, 15, 31], with one study reporting unrestricted RTA in approximately 30% of patients [31] Interestingly, active patients demonstrate better functional outcomes in terms of Constant score, ASES score, VAS for Pain and Simple Shoulder Test (*p* < 0.05) [15] A similar RTA rate after shoulder arthroplasty was reported in one study, though they assessed an older patient population with presumably lower baseline demands [31] Nonetheless, a large proportion of patients in our cohort needed to alter their primary pre‐operative sporting activity.

RTW outcomes further reinforce the functional effectiveness of RTSA. In this study, slight limitations in work ability were observed, alongside a rapid RTA. These outcomes compare favourably with others, who reported a 73.7% RTW rate in patients younger than 60 following RTSA, with lower rates among heavy labourers [30] As expected, physically demanding occupations were associated with greater post‐operative difficulty, consistent with prior reports [9, 30].

Though this study was underpowered for a subanalysis of the youngest patients in this cohort, a negative correlation between age and post‐operative SANE score was demonstrated, indicating that younger patients achieved even greater outcomes than patients approaching 65 years. This could be explained, in part, by the more severe pre‐operative pathology, such as end stage instability, that would warrant RTSA at such a young age. Furthermore, there was a slight tendency toward worse pre‐operative outcome scores in younger patients. However, this finding does provide support and optimism for the use of RTSA in even younger patients with particularly severe pathology and no other optimal treatment course.

This study has several limitations. First, its retrospective design carries an inherent risk of selection and reporting bias. Second, due to the retrospective study design, no a priori sample size calculation or power analysis was performed, as the study cohort was defined by the number of eligible patients available during the study period. Although statistically significant differences were observed in the primary outcomes, the absence of a predefined sample size calculation may limit the statistical robustness and generalisability of the findings. Third, the range of motion planes were not assessed consistently across all patients; for example, certain movements such as internal rotation and abduction were not reported in all participants, and the timing of post‐operative range‐of‐motion assessments varied. In particular, the lack of data on internal rotation and abduction—both of which are important determinants of outcomes following RTSA, may limit the strength of the presented findings. Fourth, there was substantial variability in follow‐up duration among patients, which may have influenced the reported outcomes. Also, the cohort was heterogeneous with respect to indication and prior surgical history, limiting the ability to perform diagnosis‐specific subgroup analysis. Fifth, no post‐operative radiographic evaluations were performed to assess implant positioning or radiographic changes, such as glenohumeral notching, radiographic lucency or implant loosening. Further, the questionnaires for RTA and RTW are custom‐designed and have not been validated. All procedures were performed by a single surgeon, potentially limiting the external validity and generalisability of the findings. Also, a change in implant design might have influenced the outcomes, although no differences were detected in our cohort. However, this study was not designed to compare arthroplasty designs. Finally, midterm follow‐up may be too short to adequately judge revision rates in young, active patients. Additionally, with only one failure observed, no study‐specific survivorship data could be generated, and a time‐to‐event analysis was not feasible. Therefore, no conclusions regarding survivorship can be drawn from this study.

## CONCLUSION

RTSA in patients ≤ 65 years leads to improved functional outcomes and high patient satisfaction at mid‐term follow‐up. Furthermore, it enables high rates of return to both activity and work with low rates of complication and failure.

## AUTHOR CONTRIBUTIONS

All authors contributed to the study conception and design. Material preparation, data collection, and data curation were performed by Lorenz Fritsch, Colby Wollenman, Michael Nocek, Marilee Horan, Grant Dornan, and Alan Villegas Meza. Formal analysis and software support were performed by Lorenz Fritsch, Marilee Horan and Grant Dornan. Visualisation was performed by Alan Villegas Meza. Methodology was developed by Lorenz Fritsch, Colby Wollenman, Michael Nocek, Marilee Horan, Brendon Mitchell, Grant Dornan, and Peter Millett. Validation and additional investigation were performed by Maximilian Hinz and Marco‐Christopher Rupp. Resources and supervision were provided by Peter Millett. Project administration was led by Lorenz Fritsch and Michael Nocek. The first draft of the manuscript was written by Lorenz Fritsch, Colby Wollenman, Michael Nocek, Marilee Horan, Brendon Mitchell, Grant Dornan, and Alan Villegas Meza, and all authors commented on previous versions of the manuscript. All authors read and approved the final manuscript.

## FUNDING INFORMATION

The authors have no funding to report.

## CONFLICT OF INTEREST STATEMENT

The authors declare no conflicts of interest.

## ETHICS STATEMENT

This study was performed in line with the principles of the Declaration of Helsinki. Ethical approval was obtained from the WCG Institutional Review Board (IRB) (Protocol #20252021).

## INSTITUTIONAL RELATIONSHIPS

Lorenz Fritsch, Colby Wollenman, Michael Nocek, Marilee Horan, Brendon Mitchell, Grant Dornan, Alan Villegas Meza, and Peter Millett are affiliated with the Steadman Philippon Research Institute (SPRI). SPRI is a 501(c)(3) organisation supported by philanthropy and corporate partners (including Arthrex, Canon, DJO, Icarus Medical, Medtronic, Össur, Smith+Nephew, SubioMed, Stryker, and Wright Medical). No funds, grants, or in‐kind support from these entities or any other sources were used for this study.

## DISCLOSURE

Peter Millett reports royalties, consulting, and research support from Arthrex and equity in VuMedi; these relationships are unrelated to the present work. Lorenz Fritsch, Colby Wollenman, Michael Nocek, Marilee Horan, Brendon Mitchell, Grant Dornan, Alan Villegas Meza, Maximilian Hinz, and Marco‐Christopher Rupp report no financial payments or other benefits from any commercial entity related to the subject of this article for themselves, their immediate families, or any affiliated research foundations.

## Supporting information

Supporting File 1

## Data Availability

Data available on request due to privacy/ethical restrictions.
